# A comprehensive genotype–phenotype evaluation of eight Chinese probands with Waardenburg syndrome

**DOI:** 10.1186/s12920-022-01379-6

**Published:** 2022-11-03

**Authors:** Sijun Li, Mengyao Qin, Shuang Mao, Lingyun Mei, Xinzhang Cai, Yong Feng, Chufeng He, Jian Song

**Affiliations:** 1 Department of Otorhinolaryngology, Xiangya Hospital Central South University, Changsha, Hunan China; 2 Province Key Laboratory of Otolaryngology Critical Diseases, Changsha, Hunan China; 3 Department of Otolaryngology Head and Neck Surgery, The First People’s Hospital of Changde City, Hunan Changde, China; 4 Department of Otorhinolaryngology, University of South China Affiliated Changsha Central Hospital, Changsha, Hunan China

**Keywords:** Waardenburg syndrome, Genotype, Phenotype, Inner ear malformation, Unilateral hearing loss, Heterochromia iridis

## Abstract

**Background:**

Waardenburg syndrome (WS) is the most common form of syndromic deafness with phenotypic and genetic heterogeneity in the Chinese population. This study aimed to clarify the clinical characteristics and the genetic cause in eight Chinese WS families (including three familial and five sporadic cases). Further genotype–phenotype relationships were also investigated.

**Methods:**

All probands underwent screening for the known WS-related genes including *PAX3*, *SOX10*, *MITF*, *EDNRB*, *EDN3*, and *SNAI2* using next-generation sequencing to identify disease-causing genes. Further validation using Sanger sequencing was performed. Relevant findings for the associated genotype–phenotype from previous literature were retrospectively analyzed.

**Result:**

Disease-causing variants were detected in all eight probands by molecular genetic analysis of the WS genes (*SOX10*(NM_006941.4): c.544_557del, c.553 C > T, c.762delA, c.336G > A; *MITF*(NM_000248.3): c.626 A > T; *PAX3*(NM_181459.4): c.838delG, c.452-2 A > G, c.214 A > G). Six mutations (*SOX10*:c.553 C > T, c.544_557del, c.762delA; *PAX3*: c.838delG, c.214 A > G; *MITF*:c.626 A > T) were first reported. Clinical evaluation revealed prominent phenotypic variability in these WS patients. Twelve WS1 cases and five WS2 cases were diagnosed in total. Two probands with *SOX10* mutations developed progressive changes in iris color with age, returning from pale blue at birth to normal tan. Additionally, one proband had a renal malformation (horseshoe kidneys).All cases were first described as WS cases. Congenital inner ear malformations were more common, and semicircular malformations were exclusively observed in probands with *SOX10* mutations. Unilateral hearing loss occurred more often in cases with *PAX3* mutations.

**Conclusion:**

Our findings helped illuminate the phenotypic and genotypic spectrum of WS in Chinese populations and could contribute to better genetic counseling of WS.

**Supplementary Information:**

The online version contains supplementary material available at 10.1186/s12920-022-01379-6.

## Background

Waardenburg syndrome (WS), also known as the auditory-pigmentary syndrome, is the most common cause of syndromic hearing loss (HL). It accounts for approximately 2–5% of all patients with congenital HL. There is no significant correlation between disease incidence and race or gender [1–3]. WS primarily manifests as a set of phenotypic characteristics such as sensorineural hearing loss (SNHL) and pigment abnormalities, including skin hypo-pigmentation (albinism), white forelock, premature graying of the hair, or heterochromia iridum. WS is characterized by a high phenotypic heterogeneity. This is related to the incomplete penetrance, but also to the different genes that have been identified. Depending on the clinical phenotypes, It is classified into four subtypes (I–IV), with type I and II being the most common [4]. Type I (WS1; OMIM 193500) is distinguished from type II (WS2; OMIM 193510) by the presentation of dystopia canthorum. Type III (WS3; OMIM 148820) shares similar symptoms to type I but is accompanied by upper limb abnormalities. Type IV (WS4; OMIM 277580) is type II but associated with Hirschsprung disease (HD).

To date, mutations in six genes, namely microphthalmia-associated transcription factor (*MITF*), paired box 3 (*PAX3*), endothelin 3 (*EDN3*), endothelin receptor type B (*EDNRB*), SRY Box 10 (*SOX10*), and snail homolog 2 (*SNAI2*), are responsible for WS. The molecular etiologies overlap between the subtypes *PAX3* is exclusively associated with WS1 and WS3, *MITF*, *SNAI2*, and *SOX10* are related to WS2. WS4 is mainly caused by a mutation in *SOX10*, *EDNRB*, or *EDN3*. Most of WS cases exhibit a dominant mode of inheritance and usually occur *de novo* in the probands [5]. Notably, variable phenotypes are observed within and between families, even with the same mutation making it difficult to diagnose clinically. At the molecular level, nearly 14.8% of WS1, 26.3% of WS2, and 15–35% of WS4 cases remain unexplained [6, 7].

In this study, we performed comprehensive clinical and molecular etiology analyses on eight Chinese WS families (including three familial and five sporadic cases), which led to better delineating phenotypic features and revealed several novel pathogenic mutations in *PAX3*, *SOX10*, and *MITF*. Further genotype–phenotype relationships were also investigated.

## Methods

### Patients’ description

Eight probands clinically diagnosed with WS and their family members were recruited from the Department of Otolaryngology, from the Xiangya Hospital Central South University, between January 2017 and December 2021. The study protocol was approved by the Xiangya Hospital Central South University Ethics Committee, and informed consent was obtained from each of the subject. The clinical criteria for diagnosis of WS were based on the Waardenburg Alliance, including the presence of at least two major features or one major feature plus two minor features [8].

### Clinical evaluation

A comprehensive clinical history was assessed, and audiological, neurological, ophthalmologic, and dermatologic examinations were performed on all subjects. Temporal bone high-resolution computer-assisted tomography (HRCT) and magnetic resonance imaging (MRI) were performed on the probands to evaluate the structure of the ear and brain. The audiological and neurological examinations consisted of otoscopy, pure-tone audiometry (PTA), immittance, distortion product otoacoustic emission (DPOAE), and auditory brain-stem response (ABR) tests. Additional auditory steady-state response (ASSR) tests were performed for those who did not respond to the PTA test well, due to their young age. Special attention was given to the pigmentary changes of the skin, hair, iris and other developmental defects, such as dystopia canthorum and limb abnormalities. The degree of HL was defined according to the PTA based on the three frequencies (500, 1000, and 2000 Hz). Hearing loss was categorized in the following manner: normal < 26 dB HL (decibel hearing level), mild 26–40 dB HL, moderate 41–70 dB HL, severe 71–90 dB HL, and profound > 90 dB HL. The description of the relevant inspection methods above refers to the previous studies [6, 9, 10].

### Mutation screening strategy

Blood samples were collected from all probands and some of their family members. Genomic DNA was extracted using a commercial blood DNA extraction kit following the manufacturer’s protocol (Tiangen, Beijing, China) and stored at − 80 °C until use. Target region high-throughput sequencing system (Illumina^®^ Miseq) was designed and used to screen six WS causative genes (*PAX3*, *SOX10*, *MITF*, *EDNRB*, *EDN3*, and *SNAI2*) of WS. The primer sequences for six WS-related genes were designed for the target regions as previously described [6]. Detailed methods of experiments and parameters were shown in previous studies [6]. Bidirectional sequencing validation of the target segments was performed by 2 × 250 bp sequencing with an Illumina MiSeq Sequencer by Genesky Corporation (Shanghai, China). The average effective sequencing depth for each sample was 300×, with all bases having greater than 20× sequencing depth. All pathogenic mutations detected in the cohort were validated by Sanger sequencing of patients and their family members, the primers were designed to amplify the regions flanking the variant as previously described [11].

### Mutation analysis

Bioinformatics analysis and variant interpretation were both included in Mutation analyses. The Burrows-Wheeler Aligner (http://bio-bwa.sourceforge.net/) was used to align the sequencing reads to the human reference genome (hg19); GATK Haplotype Caller software to identify the single nucleotide variants (SNVs) and short indels [12]. The nomenclature of Human Genome Variation Society guidelines (http://www.hgvs.org) was used to name the identified mutations. Mutations associated with known WS genes were detected and classified as pathogenic or likely pathogenic according to the American College of Medical Genetics and Genomics and the Association for Molecular Pathology (ACMG-AMP) guidelines [13]. The functional effects of the identified mutation were evaluated by computational tools including Polyphen-2 and MutationTaster. The conservation of the proteins was assessed among different species by ConSurf software. The sequences of these proteins were downloaded from the UniProt database (www.uniprot.org) and aligned in JalView (v2.11) software to calculate the conversation score.

## Results

### Clinical phenotypes

**Fig. 1:**
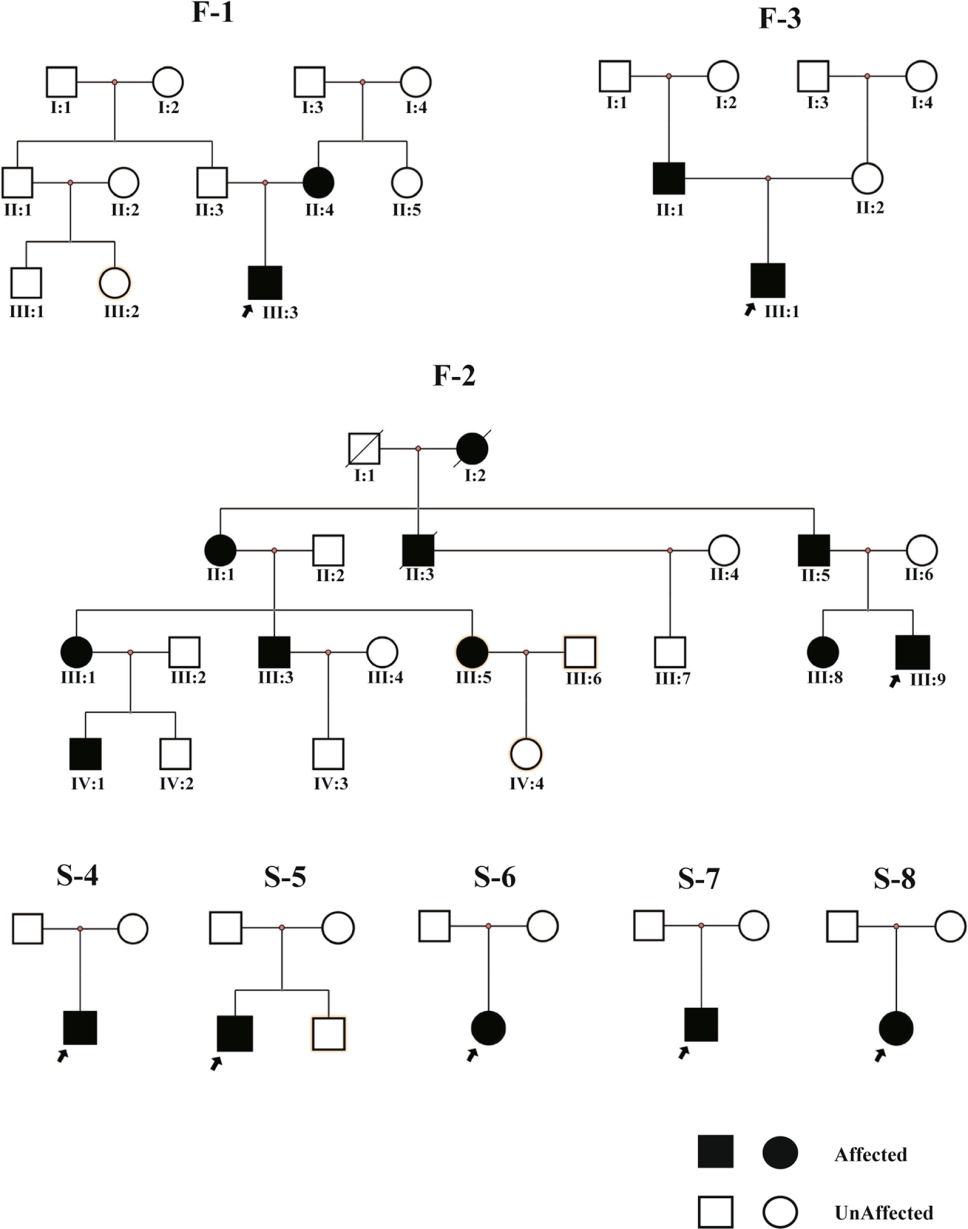
Pedigree of eight families with WS. The black arrow indicates the proband. Each generation is identified by a Roman numeral (I, II, III, IV) and each individual within the same generation is identified by an Arabic numeral (1, 2, 3 and so on). Squares represent males and circles represent females. The symbol with a diagonal line shows a deceased individual. Individuals’ symbols are colored black to indicate the presence of clinical characteristics of WS, unfilled symbols represent unaffected family members

**Fig. 2:**
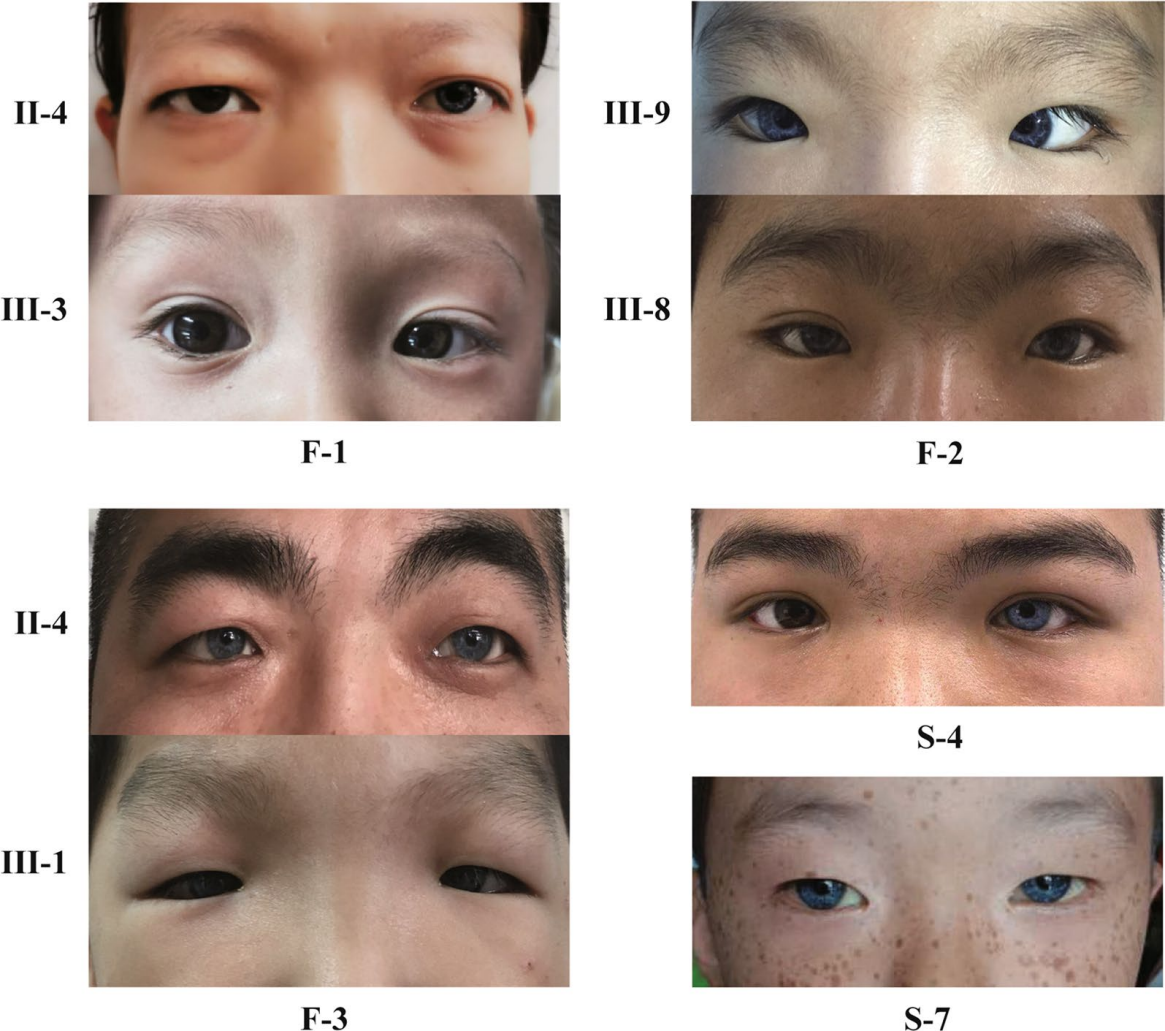
The phenotypic characteristics of WS patients included

**Fig. 3:**
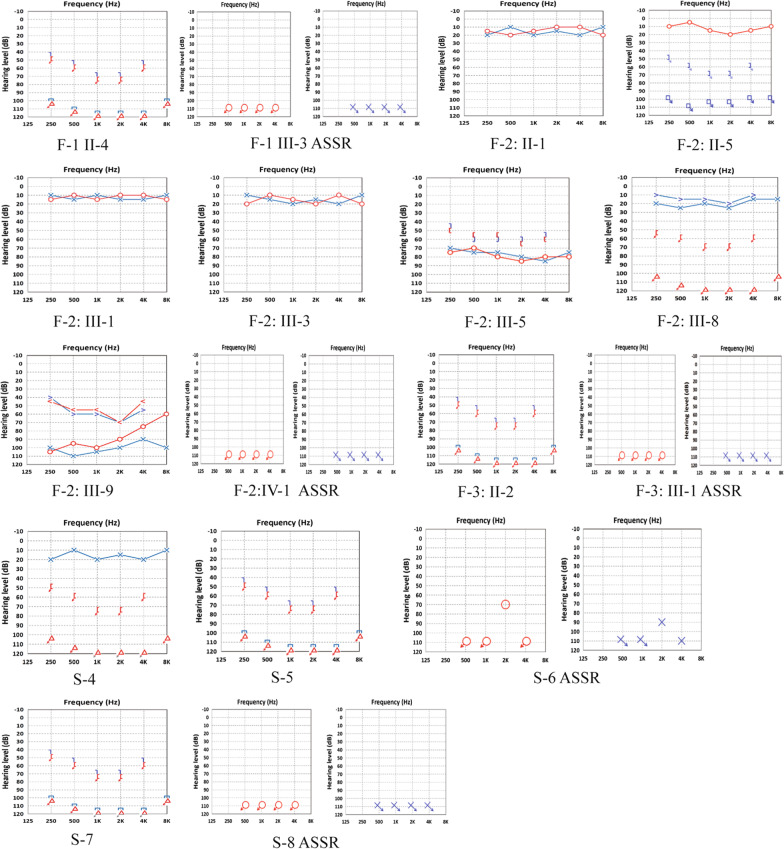
Photographs of audiogram from WS patients included

In total, 17 patients including 10 males and seven females from eight families, with some of their healthy family members, were enrolled (Fig. 1). F-2(II-5, III-9) was first described in our previous study as family 14 [6]. In this study, we expanded the collection of clinical data from other relevant patients in this extended family. The phenotypes of the related patients were recorded in detail. The study population comprised three familial and five sporadic cases ranging from one to 34 years of age. According to the diagnostic criteria of WS, five patients were diagnosed with WS2, and the others were diagnosed with WS1. The distribution of clinical features in this study population is shown in Table 1. SNHL was observed in 14 WS cases (82.4%, 14/17). The SNHL was mostly observed as bilateral non-progressive severe to profound sensorineural. However, four cases of WS1 (F2:II-5, F2:III-9, F3:II-2, S4) with unilateral SNHL were also observed, which is not common in WS cases (Figs. 2 and 3).

**Table 1:** Summary of clinical characteristics for affected WS patients

Family ID	Family member	Sex	Age	Clinical diagnosis	Severity of HL		Dystopia Canthorum	Premature white hair	Freckles	Heterochromia iridis	
					Left	Right				Left	Right
F-1	II-4	F	24	WS2	Profound	Profound	−	−	−	+	+
	III-3	M	1	WS2	Profound	Profound	−	−	−	+	+
F-2	II-1	F	56	WS1	Normal	Normal	+	−	−	+	+
	II-5	M	38	WS1	Profound	Normal	+	−	−	+	+
	III-1	F	26	WS1	Normal	Normal	+	−	−	−	+
	III-3	M	21	WS1	Normal	Normal	+	−	−	+	+
	III-5	F	20	WS1	Severe	Severe	+	−	−	+	−
	III-8	F	15	WS1	Normal	Profound	+	−	−	+	+
	III-9	M	6	WS1	Profound	Profound	+	−	−	+	+
	IV-1	M	2	WS1	Profound	Profound	+	−	−	+	+
F-3	II-2	M	34	WS1	Normal	Profound	+	−	−	+	+
	III-1	M	4	WS1	Profound	Profound	+	−	−	+	+
S-4	–	M	24	WS1	Normal	Profound	+	−	−	+	−
S-5	–	M	9	WS2	Profound	Profound	−	−	−	+	+
S-6	−	F	1	WS2	Profound	Profound	−	−	−	+	−
S-7	–	M	6	WS1	Profound	Profound	+	−	+	+	+
S-8	–	F	1.5	WS2	Profound	Profound	−	−	−	−	+

+, phenotypic presented; −, phenotypic unpresented

*F* Female;* M* Male

**Fig. 4:**
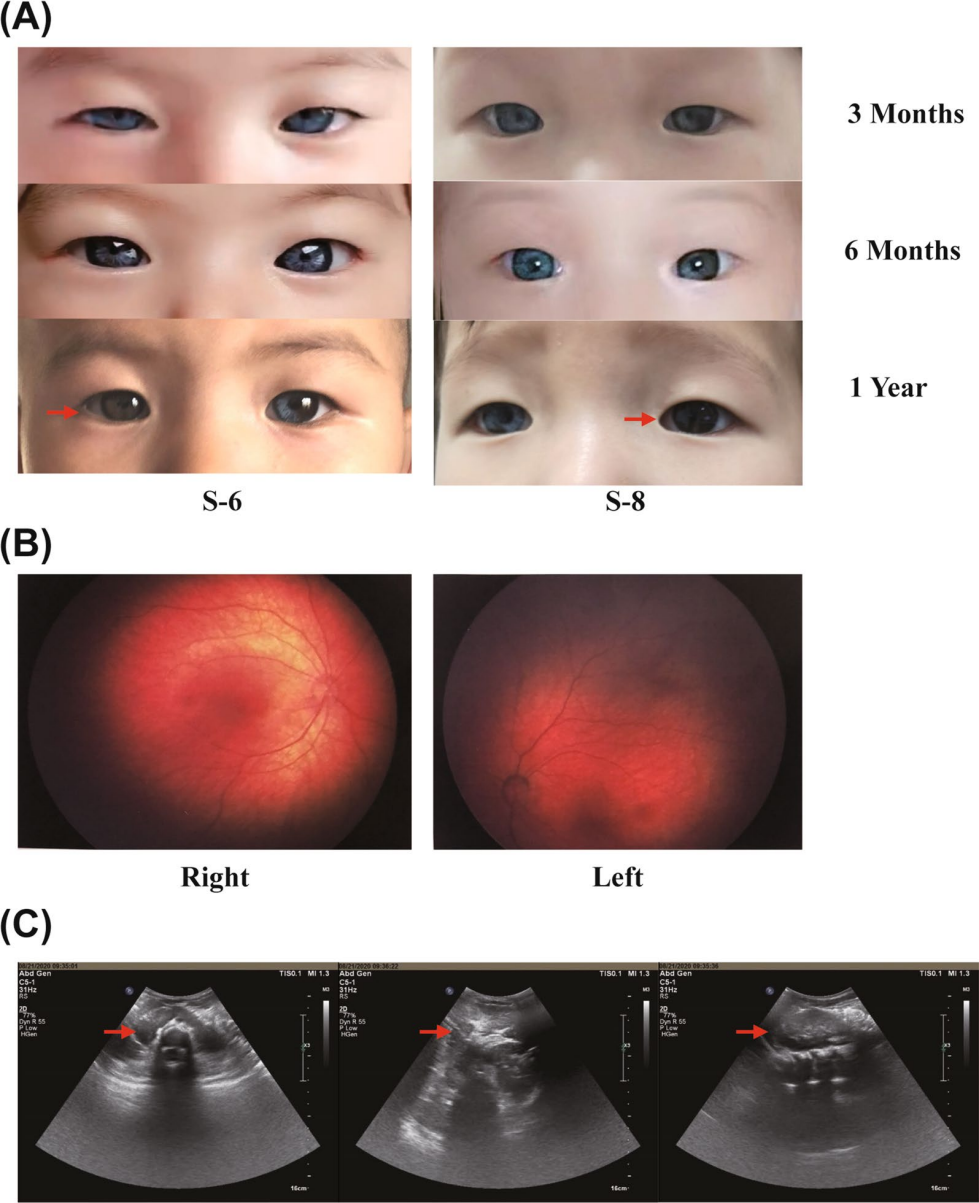
Rare clinical phenotypes of two WS2 patients. **A** Photographs of iris color of two WS2 patients at different age. **B** Retinal hypopigmentation can be seen on fundus photograph of one WS proband(S-6). **C** Renal malformation as horseshoe kidneys identified by Ultrasonography in one WS proband(S-6)

**Fig. 5:**
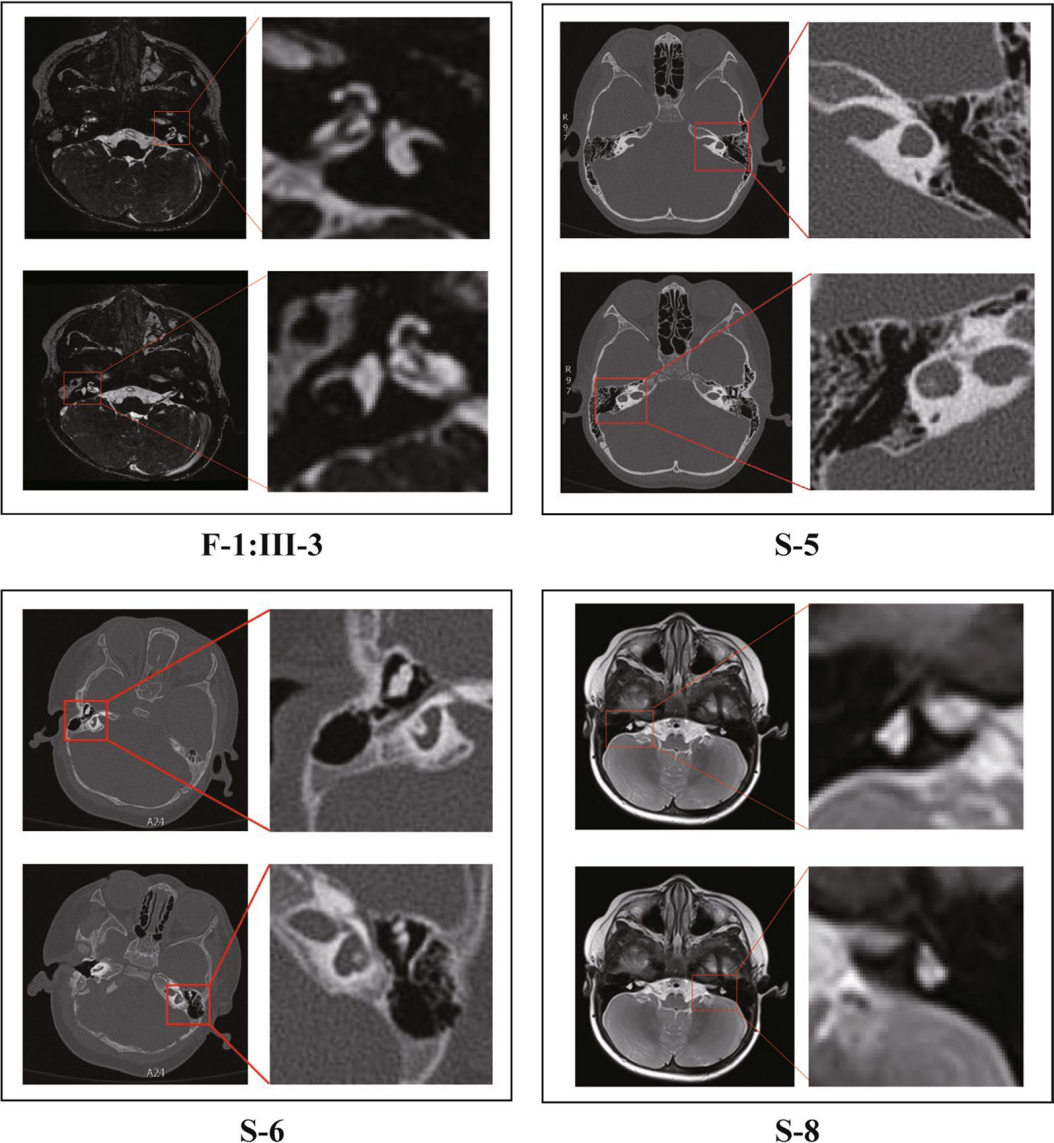
Temporal bone CT and cranial MRI of WS probands with inner ear malformation

Interestingly, two WS2 probands (S6 and S8) developed progressive changes in iris color with age, returning from pale blue (at birth) to normal tan gradually without any other pigmentation changes. Bilateral retinal hypopigmentation was also found in proband S-6 at one year of age (Fig. 4 A, B). Further, this proband (S-6) presented retinal hypopigmentation and renal malformation as horseshoe kidneys. Ultrasonography revealed deformity of the right kidney with an elongated shape, fusion occurring at the lower pole, and the isthmus connecting the two renal masses positioned at the midline. The renal function test indicated normal function (Fig. 4C). In addition, by comprehensive evaluation of Temporal bone computed tomography (CT), cranial MRI, and internal auditory canal MR hydrography, four WS2 patients (F1:III-3, S5, S6, S8) presented bilateral inner ear malformations to varying degrees (Fig. 5). One patient presented bilateral cochlea with hypoplastic middle and apical turns, dilated vestibule, and absent semicircular canal. The other three patients presented only with vestibular and semicircular hypoplasia. No abnormality of the auditory nerve was observed in any of these cases. Detailed information about imaging features is shown in Table 2.

**Table 2:** The phenotypic characteristics of inner ear malformation in WS patients

Family ID	Laterality	Cochlear	Vestibule	SSCC	LSCC	PSCC	Auditory nerve
F1:III−3	Bilaterality	Normal	Normal	Absent	Normal	Absent	Normal
S5	Bilaterality	Normal	Dilated	Normal	Hypoplasia	Normal	Normal
S6	Bilaterality	Normal	Normal	Normal	Hypoplasia	Normal	Normal
S8	Bilaterality	Hypoplasia	Dilated	Absent	Absent	Absent	Normal

*SSCC* Superior semicircular canal;* LSCC* Lateral semicircular canal;* PSCC* Posterior semicircular canal

### Spectrum of mutations

The molecular genetic analysis of the WS-related genes for eight probands identified seven heterozygous mutations in *PAX3*, *MITF*, and *SOX10* (Table 3). Three mutations occurred in the *PAX3* gene(NM_181459.4): one splice mutation c.452-2 A > G, one missense mutation c.214 A > G (p.Ile72Val), and one deletion mutation c.838delG (p.Ala280fs*4). One missense mutation c.336G > A(p.Met112Ile), one nonsense mutation c.553 C > T(p.Gln185*), and two deletion mutations c.544_557del(p.Lys182Argfs*94), c.762delA(p.Asp255Thr fs*31) were detected in the *SOX10* gene(NM_006941.4). One missense mutation c.626 A > T (p.His209Leu) was detected in the *MITF* gene(NM_000248.3). None of these eight mutations were detected in unaffected family members. The mutations detected were compared with HGMD, Exome Variant Server, and Deafness Variation databases. The results showed that six mutations have not previously been reported, excluding the c.452-2 A > G and c.336G > A variants [14–17]. Further Sanger sequencing of probands and their family members shows that all the mutations were present in the affected members. *SOX10* c.544_557del was considered *de novo* mutations by gene tests in the proband and her parents (Additional file 1: Fig. S1). In addition, all mutations were classified according to ACMG-AMP standards and guidelines for interpreting sequence variants. The mutations and pathogenicity analysis were summarized in Table 3.

**Table 3:** Genotypes of all WS probands in details and pathogenicity predictions

Family ID	Clinical Diagnosis	Inheritance status	Gene	cDNA	Protein change	Zygote	Novelty	ACMG classification	ACMG criteria
F-1	WS2	Familial	SOX10	c.553 C > T	p. Gln185*	Heterozygous	Novel	Pathogenic	PVS1 + PM2 + PP4
F-2	WS1	Familial	PAX3	c.452-2 A > G	–	Heterozygous	(Tassabehji et al.,1994)	Pathogenic	PVS1 + PM2 + PP5
F-3	WS1	Familial	PAX3	c.838delG	p. Ala280fs*4	Heterozygous	Novel	Pathogenic	PVS1 + PM2 + PP4
S-4	WS1	–	PAX3	c.214 A > G	p. Ile72Val	Heterozygous	Novel	Variants of uncertain significance	PM1 + PM2 + PP3
S-5	WS2	–	SOX10	c.336G > A	p. Met112Ile	Heterozygous	(Chaoui et al.,2011)	Likely Pathogenic	PS1 + PM2 + PP3
S-6	WS2	*De novo*	SOX10	c.544_557delAAGGCCGCCCAGGG	p.Lys182Argfs*94	Heterozygous	Novel	Pathogenic	PVS1 + PS2 + PM2
S-7	WS1	–	MITF	c.626 A > T	p. His209Leu	Heterozygous	Novel	Likely Pathogenic	PM1 + PM2 + PP3 + PM5
S-8	WS2	–	SOX10	c.762delA	p. Asp255Thr fs*31	Heterozygous	Novel	Pathogenic	PVS1 + PM2

### Genotype–phenotype correlation

The phenotypes of WS patients in this study with *PAX3, SOX10*, and *MITF* mutations are shown in Additional file 3: Table S1. To be interested, SNHL is present in 76.9% of all *PAX3* patients (10/13) and significant phenotypic differences in hearing among different individuals were observed, even within the same family. Unilateral sensorineural hearing loss (USNHL) is present in about 40% of deaf patients with *PAX3* mutations (4/10). Besides, inner ear malformations were found in 57% of the WS probands (4/7), with malformed vestibule and semicircular canals being the most common (Fig. 5). This phenotype seemed to be exclusively associated with *SOX10* variants.

## Discussion

### Mutation spectrum

**Fig. 6:**
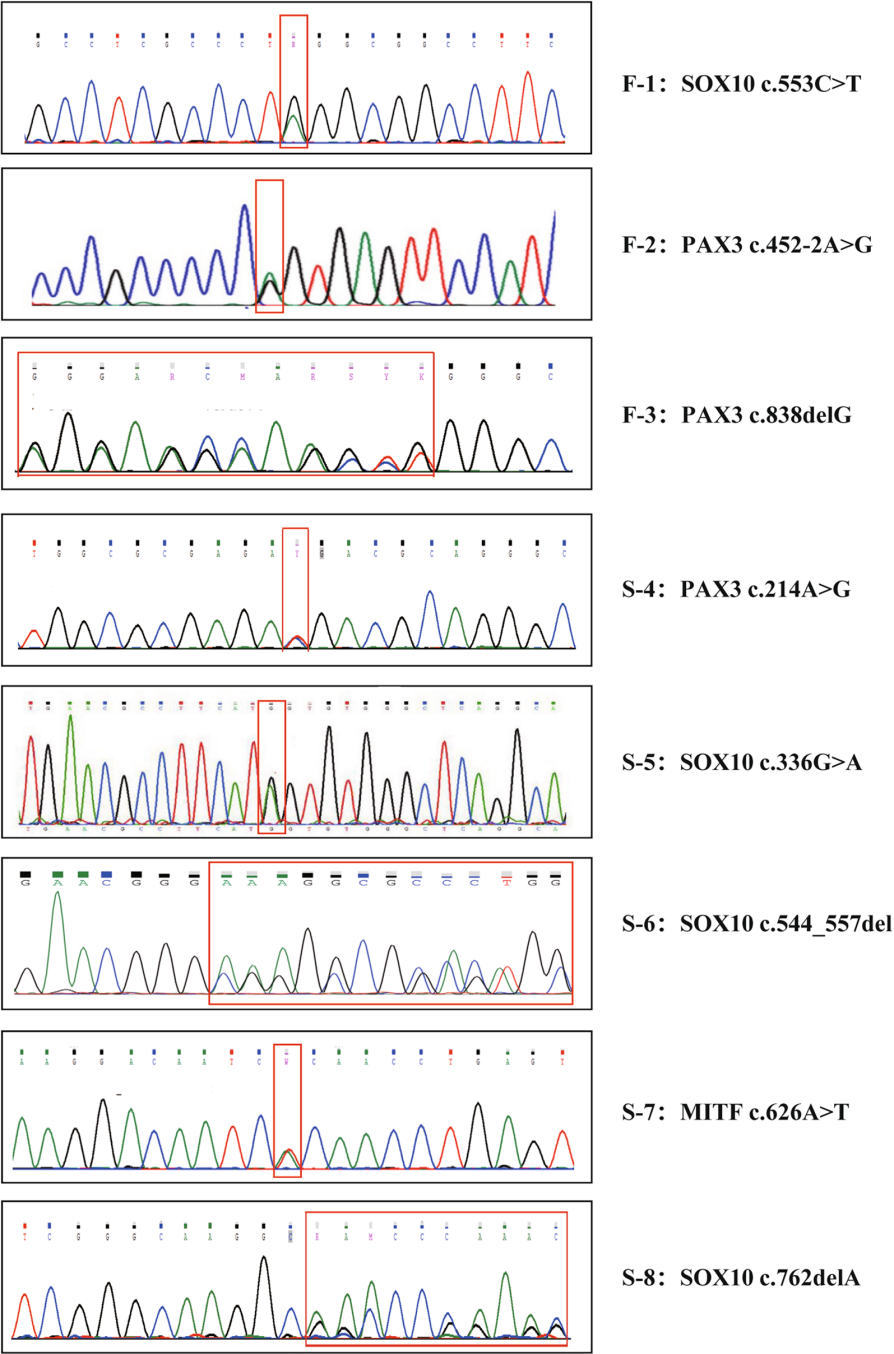
The mutated sequence identified from eight WS probands. The red box indicates the emplacement of the mutations and the change of the amino acids sequence

Molecular genetic analysis of the WS genes for eight families revealed eight heterozygous mutations (Fig. 6). Among the probands, 37.5% with WS1 (3/8, *PAX3*), 50% with WS2 (4/8, *SOX10*), and 22.5% with WS2 (1/8, *MITF*) were identified after extensive analyses of clinical phenotypes and molecular diagnosis in this cohort. No WS3 and WS4 cases or mutations in *EDNRB*, *EDN3*, or *SNAI2* were identified in our study. The heterozygous mutation *SOX10* c.553 C > T(p.Gln185*) in probands of F-1 introduced a stop codon and gave rise to a truncated *SOX10* protein with 185 of the 466 wild-type amino acids. A heterozygous mutation (*PAX3* c.452-2 A > G) in the splicing site was found in the proband, the father and sister of F-2. This mutation transformed the conservative AG sequence into AA. The mutation site would affect the normal splicing of pre-mRNA for *PAX3*, resulting in abnormal function or activity of the protein. This mutation site has been previously reported several times in different populations which could be a hot-spot mutation site of PAX3 in WS [6, 14, 15]. Therefore, we believed that the mutation is pathogenic, which should be the main pathogenic factor for the proband and his relatives of F-2. The mutations in *PAX3* c.838delG(p.Ala280fs*4) and *SOX10* c.762delA(p. Asp255Thr fs*31) were detected respectively in the probands of F-3, and S-8 caused a frameshift and stop codon into the basic domain of the corresponding protein, forming a truncated protein. These changes affected the function of the protein, resulting in the change of WS phenotype in patients. In addition, three missense heterozygote mutations *PAX3* c.214 A > G(p.Ile72Val), *SOX10*c.336G > A(p. Met112Ile) and *MITF* c.626 A > T(p.His209Leu) were detected respectively in the proband of S-4, S-5, and S-7. The *PAX3* c.214 A > G mutation resulted in the mutation of isoleucine 72 to valine. This mutation is located in the pair box domain (PD) conserved region of the PAX3 protein, and it is speculated that the change of amino acid might cause changes in the tertiary structure, affecting the protein’s function. The mutation site was conserved among multiple species and was predicted by Mutation Taster (disease-causing) and Polyphen-2 (score: 0.997). The *SOX10* c.336G > A mutation resulted in a transition from methionine to isoleucine at 112 and is located in the high-mobility group region of SOX10 protein. Chaouid et al. [17] first found the same mutation for two WS cases with deafness, which also affected the DNA binding capacity of the region. The mutation c.626 A > T in the *MITF* gene is predicted to alter the highly conserved histidine at codon 209, located in the basic helix-loop-helix leucine zipper domain [18, 19]. Therefore, this mutation might influence DNA-binding activity and transactivation capabilities, resulting in a disease phenotype predicted by Mutation Taster (disease-causing) and Polyphen-2 (score: 0.971) (Additional file 2: Fig. S2). The deletion mutation of *SOX10* c.544_557del(p.Lys182Argfs*94) in the proband of S-6 resulted in a frameshift and stop codon being inserted into the basic domain of the corresponding protein, causing a truncation. This mutation affects the function of the protein, resulting in the change of WS phenotype in patients.

### Reversed changed the color of the iris in WS

The characteristic ocular abnormality of WS is abnormal pigmentation of the iris. Changes in iris pigmentation in WS included: complete heterochromia (different colors in both eyes), partial heterochromia (fragmentary blue iris in single or both eyes), and stunted bright blue iris [20]. Previous studies have described that the incidence of iris hypopigmentation ranges from 14.9 to 42% and accounts for 47% of WS2 cases [21]. Heterochromia iridum occurred in all WS patients in the present study, and five cases showed unilateral (three WS1 with *PAX3* mutations and two WS2 with *SOX10* mutations). The pigment content of the iris substrate and the front layer determines the color of the iris. Thus, with increasing pigment in the iris substrate, there is more light absorption and a darker color in the eyes [22]. The hypopigmentation of the iris in WS has been documented on electron microscopy to manifest as fewer melanocytes in the hypopigmented region compared with the normal brown region. This results in substantial reductions in the melanosome size in the hypopigmented region and is considered to be related to a defect in neural crest cell (NCC) migration and melanin production, a process in which WS-related genes might be involved [23, 24].

In this study, two WS2 patients presented progressive iris color changes. According to the medical history and photos, the proband (S-6) was showing bilateral pale blue irises without any other symptoms of hypopigmentation when he was born. The hearing test found bilateral severe to profound hearing loss after six months. Interestingly, the color of both eyes darkened to varying degrees with age. At the time of the visit, the whole iris color of the right eye had returned to normal brown, while the other eye had a fragmentary brown iris. The other proband (S-8) shares the same medical history and symptoms. It is also worth noting that both had identified pathogenic mutations in the *SOX10* gene.

This phenomenon has not been mentioned in any other previous WS cases. Although the specific mechanism of this reversed color change in the iris is unclear, it may be related to some compensatory mechanisms of melanin. This phenomenon should attract the attention of pediatricians or otologists during clinical diagnosis and treatment. Studies have shown that variants in *SOX10* and *MITF* were identified in some nonsyndromic sensorineural hearing loss (NSHL) cases [25–27], which implies the clinical and genetic heterogeneity of this syndrome. Whether these patients may also have this reversible change in the iris needs further investigation. Altogether, the possibility of WS for these cases initially diagnosed as NSHL based on their phenotypes cannot be ruled out. We also suggest that WS-related genes, especially *SOX10* and *MITF*, should be included in clinical genetic testing for NSHL to avoid clinical misdiagnosis due to phenotypic variability.

### Heterogeneity of hearing phenotypes and PAX3 mutations

*PAX3*, also known as *HuP2*, encodes a member of the PAX family of transcription factors, characterized by a highly conserved paired DNA binding domain first identified in Drosophila [28]. It is mainly expressed in the development of neural crest(NC) derivatives, skeletal muscle, and the central nervous system, and regulates the expression of target genes that have impact proliferation, survival, differentiation, and motility [29–31]. This protein is characterized by an N-terminal DNA binding and a C-terminal transactivation domains. The DNA binding domain consists of a paired box, octapeptide, and homeodomain, and the transactivation domain contains a proline-, serine- and threonine-rich region [32]. The importance of *PAX3* in the neural tube, NC, and muscle development is confirmed by the molecular genetic findings in WS [33]. Mutations in the *PAX3* are observed in nearly 80% of WS1 cases, whereas partial or total deletion of *PAX3* and contiguous genes are often observed in WS3 cases [34]. Moreover, *PAX3* mutations have also been identified in another rare disorder called craniofacial-deafness-hand syndrome (CDHS), which is considered to be an allelic variant of Waardenburg syndrome. CDHS is characterized by craniofacial anomalies, profound sensorineural deafness, dysplasia or absence of nasal bone, slit-like nostrils, blepharophimosis, and ulnar deviation of the hand with contractures of the fingers [35, 36].

Similar to our findings, previous studies have shown heterogeneity of hearing phenotypes in WS patients with *PAX3* mutations [37, 38]. SNHL is found in 52.5% of the WS patients with *PAX3* mutation (52.3% in WS1, 57.1% in WS3), and the degree of HL differs from mild to profound, even within one family. Importantly, USNHL is more frequently associated with mutations in *PAX3* than any other WS-related genes, accounting for nearly 26% of WS1 and WS3 patients. It has also been reported that there is a strong association between the heritability of USNHL and the pigmentary abnormality[9]. Although the relevant pathogenic mechanism is still unclear, further study may be needed to focus on the effect of PAX3 during the migrating process of NCCs.

Thus far, few studies have focused on the epidemiology of USNHL. The etiology of a substantial portion of USNHL remains unknown, and genetic causes have not been elucidated. WS should be included in the differential diagnosis of children with congenital USNHL, to screen the WS-related genes, especially *PAX3*.

### Inner ear malformation and SOX10 mutations

Several recent studies have mentioned the association between inner ear malformations and *SOX10* mutations [7, 39–41]. Bilateral hypoplasia or dysplasia of the semicircular canals and enlarged vestibules are very frequent, while agenesis of the vestibulo-cochlear nerve and cochlear deformities have also been reported. These previous reports are somewhat consistent with our findings.

To further investigate the genotype–phenotype relationship between *SOX10* mutations and inner ear malformation, an up-to-date literature overview was performed (Additional file 4: Table S2). WS patients with *SOX10* mutation and inner ear malformations from the previous report were included. 60 individual WS patients were found to meet this inclusion criterion. Gender distribution was 23.8% female versus 76.2% male persons (gender was provided in 21 patients) and the mean age of the included patients was 5.6 years (standard deviation 7.9, exact age was provided in 37 patients). Fifty-six of them are WS2 and others are WS4. Temporal bone abnormalities were mostly bilateral (57/60). The morphologic abnormalities of the vestibular were shown in 96.7% (58/60) of the *SOX10*-related WS cases. The most frequently reported vestibular aberrations were agenesis or hypoplasia of ≥ 1 semicircular canals (SCC) (92.8%, 39/42), agenesis of posterior SCC was seen with the highest frequency (85.7%, 36/42) followed by the lateral SCC (83.3%, 35/42) and superior SCC (66.7%, 28/42). Enlarged or malformed vestibules were also observed frequently (76.2%, 32/42). Meanwhile, 76.7% (46/60) of the patients presented with the hypoplastic cochlea, which mainly showed reduced size, hypoplastic middle, and apical turns with a short modiolus. The cochlear nerves were bilaterally absent in five of the six patients who underwent MRI and were unilaterally absent in one patient. These patients have various types of mutations, including nonsense, missense, deletion, and frameshift. The copy number variations were also observed. However, within these *SOX10*-related WS cases, we did not find a correlation between the mutation and the type or severity of the inner ear malformations observed.

SOX10 belongs to the SOX family of transcription factors, mainly involved in developmental skeletogenesis, neurogenesis, and NC development [42, 43]. It is vital in promoting cell survival, maintaining multipotency of NC stem cells, and controlling cell fate for various NC derivatives [24].

So far, the pathogenic mechanism of inner ear malformation caused by a mutated SOX10 protein is still not fully understood. In melanocytes, SOX10 manipulates proliferation, survival, and differentiation by activating downstream target genes, like *MITF*. Mutations in *SOX10* may result in a developmental defect in melanocyte-derived cell lineage of the inner ear, called intermediate cells of the stria vascularis, necessary for inner ear homeostasis, and eventually cause HL. Unlike the other pathogenic genes of WS, *SOX10* is expressed early in the otic vesicle from four weeks of human embryonic development and then in the developing epithelium of the cochlea and vestibule, before being restricted to supporting cells of the neurosensory epithelium. It promotes the survival of cochlear progenitors during the formation of the otocyst and the organ of Corti [44]. It is also expressed in the glial development of the cochleovestibular ganglia[45]. Breuskin et al. [46] demonstrated that loss of *Sox10* function decreases the expression of *Jag1*, which plays an important role in the maintenance and differentiation of inner ear sensory epithelial progenitors, mediate by the Notch pathway and thus leads to the malformation of the inner ear during the development of Xenopus. Hao et al. [47] identified some key genes (*WNT1*, *KCNQ4*, *STRC*, and *PAX6*) and pathways associated with inner ear malformation in a pig model with *SOX10* mutations, through RNA-seq analysis. Wen et al. [48] established WS patient-derived induced pluripotent stem cells and differentiated them into NCCs. RNA-seq identified a cluster of differentially expressed genes (DEGs) associated with inner ear development and morphogenesis, providing a rich context for investigating the molecular etiology of WS regarding inner ear malformations.

### Waardenburg syndrome with renal involvement

Few studies have mentioned renal involvement in sporadic cases of WS, which includes duplicated ureteral collecting system, multicystic dysplastic kidney, and horseshoe kidney [49–51]. One WS family with three generations has renal agenesis which makes this finding likely to be hereditary [52]. However, no correlation between the genotype and associated phenotype can be found. A recent study has shown that Mitf-M-null mice have enlarged kidneys indicating a role for *MITF-M* in size control of the kidney [53]. To our knowledge, there has been no mention elsewhere in previous studies of *SOX10* mutation with associated renal anomalies. The *SOX10* gene is a characteristic marker for multipotent NC progenitors for various NC derivatives. During the development of the urinary system, NCC migrates to the embryologic kidney and forms nephrogenic mesenchyme, which induces differentiation and formation of the kidney [54]. Congenital kidney malformations can occur from aberrant NCC, as evidenced in previous reports. Collectively, whether the renal abnormality is a symptom of WS still needs to be further investigated.

## Conclusion

In conclusion, this study presented a comprehensive genetic and clinical investigation of WS in a group of Chinese patients. Several unusual phenotypes were first found in WS cases, which should also be considered in the wide spectrum of WS. In addition, six novel heterozygous mutations were identified, which expanded the database of unknown mutations in WS patients. Moreover, our data revealed the clinical and genetic spectra and particular genotype–phenotype relationships in a Chinese population, which expanded our understanding of WS.

## Supplementary Information

**Additional file 1: Fig. S1.** **The mutated sequence identified from the S-6 proband with his parents.** The red box indicates the emplacement of the mutations and the change of the amino acids sequence.

**Additional file 2: Fig. S2.** **The schematic representation and protein conservativeness analysis of the localization of two new missense mutations.** A. The PAX3 gene mutation detected in the S-4.The variant c.214A>G(p.Ile72Val) represented in red is a novel mutation.The mutation site was conservative among multiple species. PBD: pair box domain;HD: HMG domain. B. The MITF gene mutation detected in the S-7.The variant c.626A>T(p.His209Leu) represented in red is a novel mutation.The mutation site was conservative among multiple species. Basic: Basic domain; HLH: Helix-Loop-Helix domain; LZ: Leucine zipper domain.

**Additional file 3: Table S1.** **Phenotypes in WS patients with PAX3, SOX10 and MITF mutations**.

**Additional file 4: Table S2.** **Clinical phenotypes and genotypes of the SOX10 mutated patients with inner ear malformations**.

## Data Availability

The datasets generated and/or analysed during the current study are available in Leiden Open Variation Database (LOVD; https://databases.lovd.nl/shared/; Individual ID: #00408235, #00408236, #00415811, #00415812, #00415813, #00415814, #00415815, #00415816). The raw sequence data reported in this study have been deposited in the SRA databases with the accession number PRJNA887200 (https://www.ncbi.nlm.nih.gov/sra/PRJNA887200).

## Abbreviations

WS: Waardenburg syndrome
HL: Hearing loss
SNHL: Sensorineural hearing loss
HD: Hirschsprung disease
*MITF*: Microphthalmia-associated transcription factor
*PAX3*: Paired box 3
*EDN3*: Endothelin 3
*EDNRB*: Endothelin receptor type B
*SOX10*: SRY Box 10
*SNAI2*: Snail homolog 2
HRCT: Temporal bone high-resolution computer-assisted tomography
MRI: Magnetic resonance imaging
PTA: Pure-tone audiometry
DPOAE: Distortion product otoacoustic emission
ABR: Auditory brain-stem response
ASSR: Auditory steady-state response
SNVs: Single nucleotide variants
ACMG-AMP: American College of Medical Genetics and Genomics and the Association for Molecular Pathology
USNHL: Unilateral sensorineural hearing loss
NSHL: Nonsyndromic sensorineural hearing loss
NCC: Neural crest cell
CDHS: Craniofacial-deafness-hand syndrome
SCC: Semicircular canals
SSCC: Superior semicircular canal
LSCC: Lateral semicircular canal
PSCC: Posterior semicircular canal
DEGs: Differentially expressed genes
