# Supplementary material for: A comprehensive genotype–phenotype evaluation of eight Chinese probands with Waardenburg syndrome
Source: BMC Med Genomics. 2022 Nov 3;15:230. doi: 10.1186/s12920-022-01379-6 (PMC9632049; doi:10.1186/s12920-022-01379-6)

**Supplementary Figure1. The mutated sequence identified from the S-6 proband with his parents**. The red box indicates the emplacement of the mutations and the change of the amino acids sequence.

## S-6：SOX10 c.544_557del

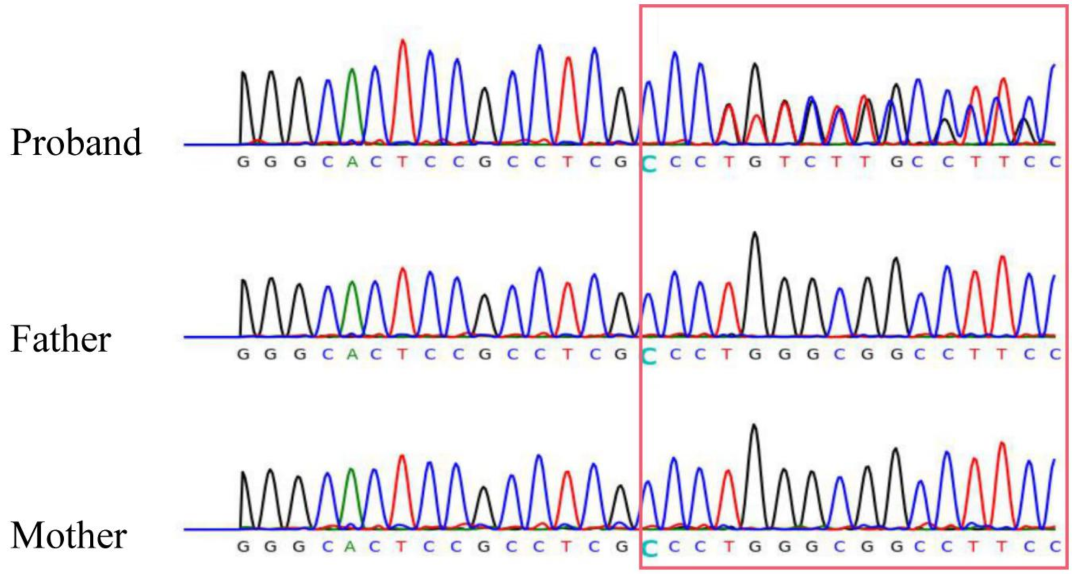

Supplement: Supplementary file 1 — Additional file 1: Fig. S1. The mutated sequence identified from the S-6 proband with his parents. The red box indicates the emplacement of the mutations and the change of the amino acids sequence. [file 12920_2022_1379_MOESM1_ESM.pdf]
