# Supplementary material for: A comprehensive genotype–phenotype evaluation of eight Chinese probands with Waardenburg syndrome
Source: BMC Med Genomics. 2022 Nov 3;15:230. doi: 10.1186/s12920-022-01379-6 (PMC9632049; doi:10.1186/s12920-022-01379-6)

Supplementary Figure2. The schematic representation and protein conservativeness analysis of the localization of two new missense mutations.

A. The PAX3 gene mutation detected in the S-4.The variant c.214A>G(p.Ile72Val) represented in red is a novel mutation.The mutation site was conservative among multiple species. PBD: pair box domain; HD: HMG domain.

B. The MITF gene mutation detected in the S-7.The variant c.626A>T(p.His209Leu) represented in red is a novel mutation.The mutation site was conservative among multiple species. Basic: Basic domain; HLH: Helix-Loop-Helix domain; LZ: Leucine zipper domain.

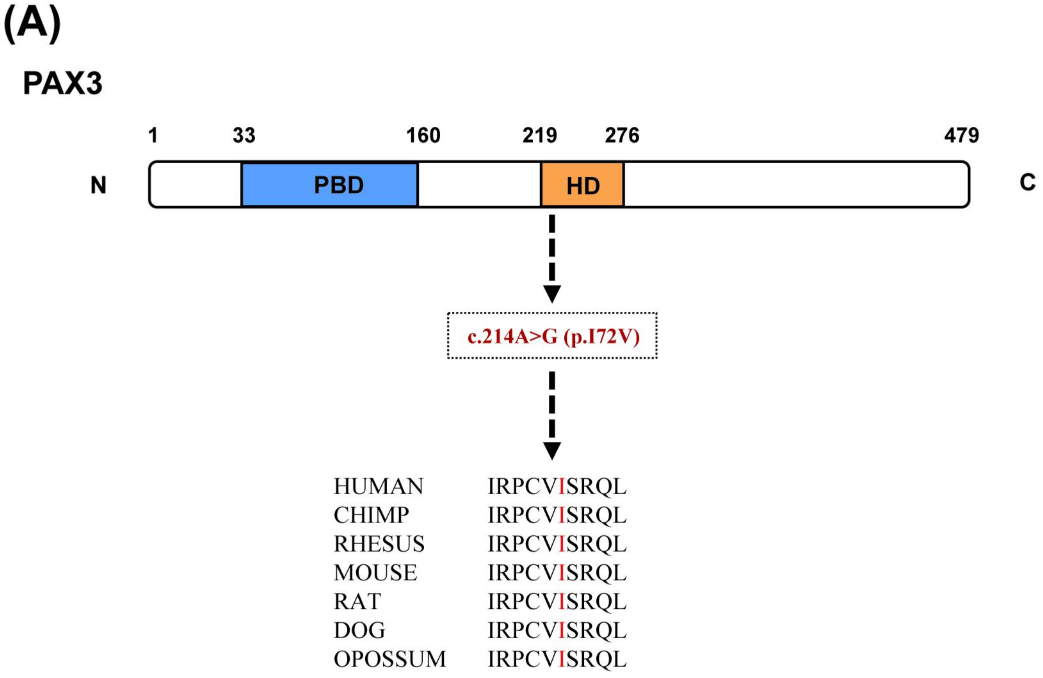

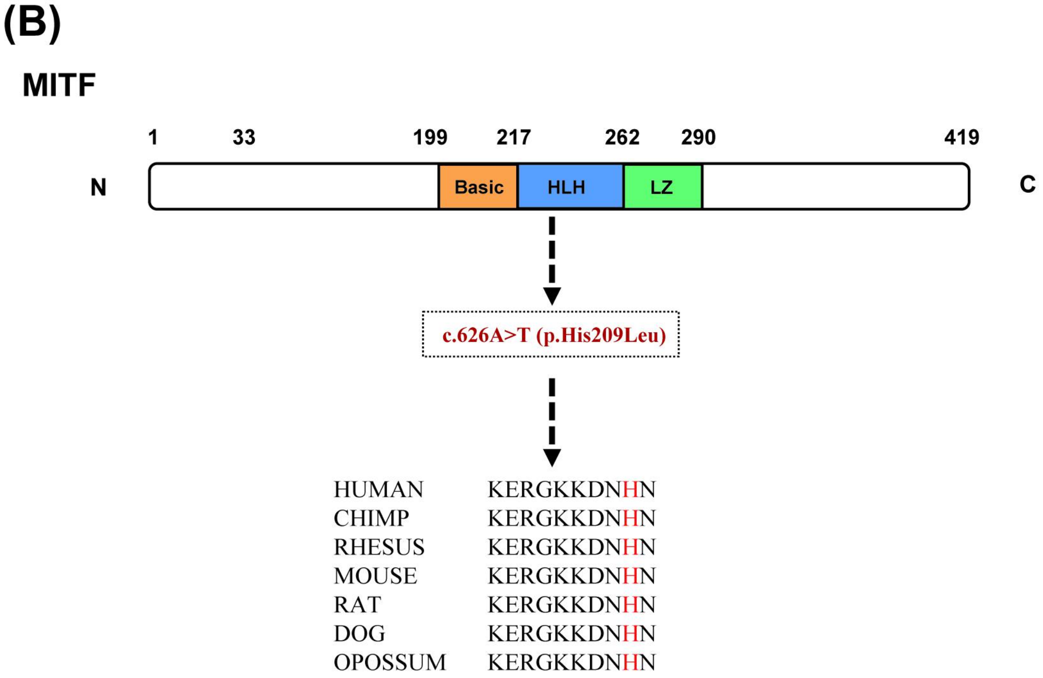

Supplement: Supplementary file 2 — Additional file 2: Fig. S2. The schematic representation and protein conservativeness analysis of the localization of two new missense mutations. A. The PAX3 gene mutation detected in the S-4.The variant c.214A>G(p.Ile72Val) represented in red is a novel mutation.The mutation site was conservative among multiple species. PBD: pair box domain;HD: HMG domain. B. The MITF gene mutation detected in the S-7.The variant c.626A>T(p.His209Leu) represented in red is a novel mutation.The mutation site was conservative among multiple species. Basic: Basic domain; HLH: Helix-Loop-Helix domain; LZ: Leucine zipper domain. [file 12920_2022_1379_MOESM2_ESM.pdf]
