# Supplementary material for: A comprehensive genotype–phenotype evaluation of eight Chinese probands with Waardenburg syndrome
Source: BMC Med Genomics. 2022 Nov 3;15:230. doi: 10.1186/s12920-022-01379-6 (PMC9632049; doi:10.1186/s12920-022-01379-6)
Supplement: Supplementary file 3 — Additional file 3: Table S1. Phenotypes in WS patients with PAX3, SOX10 and MITF mutations. [file 12920_2022_1379_MOESM3_ESM.docx]

**Supplementary Table 1. Phenotypes in WS patients with PAX3, SOX10 and MITF mutations.**

| **Phenotypes** | **PAX3** | **SOX10** | **MITF** |
| --- | --- | --- | --- |
| Hearing loss | 9 | 4 | 1 |
| Blue iris | 12 | 4 | 1 |
| Abnormal hair pigmentation | 0 | 0 | 0 |
| Freckles | 0 | 0 | 1 |
| Dystopia Canthorum | 12 | 0 | 0 |
