# Supplementary material for: A comprehensive genotype–phenotype evaluation of eight Chinese probands with Waardenburg syndrome
Source: BMC Med Genomics. 2022 Nov 3;15:230. doi: 10.1186/s12920-022-01379-6 (PMC9632049; doi:10.1186/s12920-022-01379-6)
Supplement: Supplementary file 4 — Additional file 4: Table S2. Clinical phenotypes and genotypes of the SOX10 mutated patients with inner ear malformations. [file 12920_2022_1379_MOESM4_ESM.pdf]

Supplementary Table 2. Clinical phenotypes and genotypes of the SOX10 mutated patients with inner ear malformations

Number	WS type	Mutation	Sex	Age	Laterality	Cochlear	Vestibule	SSCC	LSCC	PSCC	Auditory nerve	Reference
1	2	c.246delC.(p.Giy82GlyfsX27)	Male	5Y	Bilaterality	Normal	Dilated	Absent	Hypoplasia	Absent	Normal	(Chen et al. 2020)
2	2	c.1107delC(p.Tyr369)	Male	12Y	Bilaterality	Normal	Hypoplasia	Normal	Normal	Normal	Normal	(Burke et al. 2020)
3	2	c.259-260delCT (p.L87del)	Male	5Y	Bilaterality	Normal	Normal	Normal	Enlarged	Hypoplasia	Normal	(Burke et al. 2020)
4	4	c.652G＞T（p.G218）	Male	5M	Bilaterality	Hypoplasia	Normal	Hypoplasia	Hypoplasia	Hypoplasia	Normal	(Suzuki et al. 2018)
5	2	c.1114C>T(p.Q372X)	Male	14M	Bilaterality	Normal	Normal	Hypoplasia	Hypoplasia	Hypoplasia	Normal	(Pingault et al. 2002)
6	4	g.(17,712,505_17,716,229)_(17,929,647_17,933,832) del	Male	36Y	Bilaterality	Normal	Dilated	Normal	Hypoplasia	Hypoplasia	Normal	(Bondurand et al. 2007)
7	2	c.219_428+43del	Male	9Y	Bilaterality	Normal	Hypoplasia	Normal	Hypoplasia	Hypoplasia	Normal	(Bondurand et al. 2007)
8	4	c.915delG(p.His306ThrfsX5)	Male	9Y	Bilaterality	Hypoplasia	Normal	Normal	Normal	Normal	Normal	(Viñuela et al. 2009)

9	4	c.698-2A > C	Male	29M	Bilaterality	Normal	Dilated	Absent	Absent	Absent	Normal	(Sznajer et al. 2008)
10	2	c.521A>C(Q174P)	Male	17M	Bilaterality	Hypoplasia	Normal	Normal	Normal	Normal	Absent	(Barnett et al. 2009)
11	4	c.126_127delGCinsCT(p.Arg43X)	Male	-	Bilaterality	Hypoplasia	Enlarged	Normal	Large arch, thin	Hypoplasia	Normal	(Elmaleh-Bergès et al. 2013)
12	4	c.391A>C(p.Asn131His)	Male	32M	Bilaterality	Small	Enlarged	Large arch, thin	Large arch, thin	Small arch, thick	Normal	(Elmaleh-Bergès et al. 2013)
13	2	c.398A>G(p.Glu133Gly)	Male	32M	Bilaterality	Small	Enlarged	Normal	Small arch, thick	Small arch, thick	Normal	(Elmaleh-Bergès et al. 2013)
14	4	c.519C>G(p.Tyr173X)	Male	1M	Bilaterality	Hypoplasia	Enlarged	Small arch, thick	Small arch, thick	Small arch, thick	Normal	(Elmaleh-Bergès et al. 2013)
15	4	c.519C>G(p.Tyr173X)	Male	2M	Bilaterality	Hypoplasia	Enlarged	R: small arch, thick<br>L: Normal	Small arch, thick	Normal	Normal	(Elmaleh-Bergès et al. 2013)
16	4	c.644_648delGGCAC(p.Arg215ProfsX64)	Male	3Y	Bilaterality	Hypoplasia	Enlarged	Hypoplasia	Hypoplasia	Hypoplasia	Normal	(Elmaleh-Bergès et al. 2013)
17	4	c.700C>T (p.Gln234X)	Male	2M	Bilaterality	Hypoplasia	Enlarged	Hypoplasia	Hypoplasia	Hypoplasia	Absent	(Elmaleh-Bergès et al. 2013)

18	4	c.811delA(p.Ile271SerfsX15)	Male	5Y	Bilaterality	Hypoplasia	Enlarged	Hypoplasia	Hypoplasia	Hypoplasia	Normal	(Elmaleh-Bergès et al. 2013)
19	2	c.921delA(p.Gly308AlafsX3)	Female	18Y	Bilaterality	Hypoplasia	Enlarged	Hypoplasia	Small, thin	Hypoplasia	R: Normal; L: present, thin	(Elmaleh-Bergès et al. 2013)
20	4	c.1040delC(p.Pro347HisfsX10)	Female	20M	Bilaterality	Hypoplasia	Enlarged	Small arch, thick	small,thin	Hypoplasia	Normal	(Elmaleh-Bergès et al. 2013)
21	4	c.1058delA(p.Ala354CysfsX2)	Male	4Y	Bilaterality	Hypoplasia	Enlarged	Hypoplasia	Hypoplasia	Hypoplasia	R: present,thin; L: absent	(Elmaleh-Bergès et al. 2013)
22	4	c.1114C>T(p.Gln372X)	Male	18M	Bilaterality	Normal	Enlarged	Hypoplasia	Small, thin	Hypoplasia	Normal	(Elmaleh-Bergès et al. 2013)
23	4	c.1195_1196delCA(p.Gln399ValfsX2)	Male	16Y	Bilaterality	Small	R: enlarged; L: N	Hypoplasia	Small arch, thick	Small arch, thick	N/A	(Elmaleh-Bergès et al. 2013)
24	4	c.1401A>C(p.*467TyrextX86)	Female	8D	Bilaterality	Hypoplasia	Enlarged	Hypoplasia	Hypoplasia	Hypoplasia	Absent	(Elmaleh-Bergès et al. 2013)
25	4	Full gene deletion(p.0?)	Male	31Y	Bilaterality	Hypoplasia	Enlarged	Small arch, thick	Small arch, thick	Hypoplasia	Normal	(Elmaleh-Bergès et al. 2013)

26	2	c.621C > A(p.Y207X)	Male	5Y	Bilaterality	Hypoplasia	Normal	Hypoplasia	Hypoplasia	Hypoplasia	Normal	(Chen et al. 2015)
27	4	c.842dupT	Female	13D	Bilaterality	Hypoplasia	Normal	Hypoplasia	Hypoplasia	Hypoplasia	Normal	(Akutsu et al. 2018)
28	2	c. 115G>T (p.G39X)	Female	3Y	Bilaterality	Hypoplasia	Enlarged	Enlarged	Enlarged	Absent	Present, thin	(Arimoto et al. 2014)
29	2	c.355C > T(Arg119Cys)	Male	10M	Bilaterality	Small	Enlarged	Small arch, thick	Absent	Absent	Normal	(Niu et al. 2021)
30	2	c.235delC (p.L79Cfs*3)	Female	7Y	Bilaterality	Normal	Normal	Normal	Normal	Enlarged	Normal	(Yu et al. 2020)
31	2	c.336G>A（p.Met112Ile）	Male	4Y	Bilaterality	Normal	Dilated	Normal	Dilated	Normal	Normal	(Pingault et al. 2014)
32	2	c.7G>T（p.Glu3X）	-	-	Bilaterality	Hypoplasia	Hypoplasia	-	-	-	Normal	(Wang et al. 2021)
33	2	c.127C>T（p.Arg43X）	-	-	Bilaterality	Hypoplasia	Hypoplasia	-	-	-	Normal	(Wang et al. 2021)
34	2	c.232C>T（p.Gln78X）	-	-	Bilaterality	Hypoplasia	Hypoplasia	-	-	-	Normal	(Wang et al. 2021)
35	2	c.323T>C（p.Met108Thr）	-	-	Bilaterality	Normal	Hypoplasia	-	-	-	Normal	(Wang et al. 2021)

36	2	c.326A>G（p.Asn109Ser）	-	-	Bilaterality	Hypoplasia	Hypoplasia	-	-	-	Normal	(Wang et al. 2021)
37	2	c.335T>G（p.Met112Arg）	-	-	Bilaterality	Hypoplasia	Hypoplasia	-	-	-	Normal	(Wang et al. 2021)
38	2	c.341G>A（p.Trp114X）	-	-	Bilaterality	Normal	Hypoplasia	-	-	-	Normal	(Wang et al. 2021)
39	2	c.386T>C（p.Leu129Pro）	-	-	Bilaterality	Normal	Hypoplasia	-	-	-	Normal	(Wang et al. 2021)
40	2	c.424T>C（p.Trp142Arg）	-	-	Bilaterality	Hypoplasia	Hypoplasia	-	-	-	Normal	(Wang et al. 2021)
41	2	c.428+1G>A	-	-	Bilaterality	Hypoplasia	Hypoplasia	-	-	-	Normal	(Wang et al. 2021)
42	2	c.448A>T（p.Lys150X）	-	-	Bilaterality	Hypoplasia	Hypoplasia	-	-	-	Normal	(Wang et al. 2021)
43	2	c.463G>T（p.Glu155X）	-	-	Bilaterality	Hypoplasia	Hypoplasia	-	-	-	Normal	(Wang et al. 2021)
44	2	c.523C>T（p.Prp175Ser）	-	-	Bilaterality	Hypoplasia	Hypoplasia	-	-	-	Normal	(Wang et al. 2021)
45	2	c.776_780delACGGG（p.Asp259AlafsX20）	-	-	Bilaterality	Hypoplasia	Hypoplasia	-	-	-	Normal	(Wang et al.

												2021)
46	2	c.1063C>T（p.Gln355X）	-	-	Bilaterality	Hypoplasia	Hypoplasia	-	-	-	Normal	(Wang et al. 2021)
47	2	c.1195C>T（p.Gln399X）	-	-	Bilaterality	Hypoplasia	Hypoplasia	-	-	-	Normal	(Wang et al. 2021)
48	2	c.1352_1359dup（p.His454fsX51）	-	-	Bilaterality	Hypoplasia	Hypoplasia	-	-	-	Normal	(Wang et al. 2021)
49	2	Exon 1-4 deletion	-	-	Bilaterality	Hypoplasia	Hypoplasia	-	-	-	Normal	(Wang et al. 2021)
50	2	c.259-260delCT	Male	6Y	Bilaterality	Normal	Normal	Normal	L:Enlarged：R:Normal	Hypoplasia	Normal	(Chen et al. 2014)
51	2	c.445A>T(P.Lys149X)	Male	-	Bilaterality	Hypoplasia	Enlarged	Absent	Hypoplasia	Absent	Normal	(Dong 2014)
52	2	c.463G>T(P.Glu155X)	Female	-	Bilaterality	Hypoplasia	Enlarged	Absent	Hypoplasia	Absent	Normal	(Dong 2014)
53	2	c.463-464delGA	Female	-	Bilaterality	Hypoplasia	Enlarged	Absent	Small	Small	Normal	(Dong 2014)
54	2	c.697G>A(P.Gly233Ser)	Male	-	Bilaterality	Hypoplasia	Enlarged	Normal	Hypoplasia	Normal	Normal	(Dong 2014)
55	2	c.50_53insGTCT(P.S17fsX50)	Female	19M	Bilaterality	Hypoplasia	Enlarged	Absent	Absent	Absent	Normal	(Xu et al. 2016)
56	2	c.463G>T(P.E155X)	Male	15M	Bilaterality	Hypoplasia	Enlarged	Normal	Normal	Absent	Normal	(Xu et al. 2016)

57	2	c.1100delG(P.P367fs)	Female	1Y	Bilaterality	Hypoplasia	Enlarged	Absent	Absent	Absent	Normal	(Xu et al. 2016)
58	2	c.455_456delCC(P.P152fsX5)	Female	13M	Bilaterality	Hypoplasia	Enlarged	Absent	Absent	Absent	Normal	(Xu et al. 2016)
59	2	c.IVS2-2A>T	Male	7Y	Bilaterality	Hypoplasia	Enlarged	Normal	Absent	Absent	Normal	(Xu et al. 2016)
60	2	C.448A>T(P.K150X)	Male	28M	Bilaterality	Hypoplasia	Enlarged	Absent	Normal	Absent	Normal	(Xu et al. 2016)

D: Days M: Months Y:years; SSCC: Superior semicircular canal; LSCC:Lateral semicircular canal; PSCC: Posterior semicircular canal; R:Right side; L:Left side
